# Environmental Impact of the Hungarian Swine Sector during the PRRS Eradication Program with Full Herd Replacement (2014–2022)

**DOI:** 10.3390/ani14202924

**Published:** 2024-10-11

**Authors:** László Búza, István Szabó, László Gombos, László Varga, Veronika Szűr-Gombos, István Szabó

**Affiliations:** 1 Institute of Food Chain Science, University of Veterinary Medicine, 2 István Street, 1078 Budapest, Hungary; laszlo.buza@yahoo.com; 2 Department of Environmental Toxicology, Hungarian University of Agriculture and Life Sciences, 1 Páter Károly Street, 2100 Gödöllő, Hungary; szabo.istvan.temi@uni-mate.hu; 3 Wittmann Antal Multidisciplinary Doctoral School of Plant, Animal, and Food Sciences, Széchenyi István University, 2 Vár Square, 9200 Mosonmagyaróvár, Hungary; 4 Department of Food Science, Széchenyi István University, 15-17 Lucsony Street, 9200 Mosonmagyaróvár, Hungary; varga.laszlo@sze.hu; 5 Vadócdoki Research and Development Division, 75 Gébárti Street, 8900 Zalaegerszeg, Hungary; szurve@gmail.com; 6 National PRRS Eradication Committee, 24 Keleti Károly Street, 1024 Budapest, Hungary; iszabodr@t-online.hu

**Keywords:** porcine reproductive and respiratory syndrome, swine, sustainability, carbon footprint, carbon dioxide equivalent

## Abstract

**Simple Summary:**

The PRRS eradication program in Hungary, carried out from 2014 to 2022, focused on replacing entire herds and using high-performance breeds to improve pig farming. This approach reduced the sow population by over 26% while keeping nearly the same number of pigs for slaughter. As a result, there were significant reductions in harmful emissions, such as ammonia and greenhouse gases, and a decrease in feed and water usage. The results show that tailored strategies and advanced breeding can make pig farming more efficient and environmentally friendly. This highlights the importance of collaboration between scientists, policymakers, and industry to create sustainable livestock practices.

**Abstract:**

The Porcine Reproductive and Respiratory Syndrome (PRRS) eradication program in Hungary, implemented between 2014 and 2022, utilized complete herd replacement and the introduction of high-performance breeds to enhance production efficiency and environmental sustainability in the swine sector. As a result, the sow population was reduced by 26.2% while maintaining nearly the same number of slaughter pigs. This led to significant reductions in ammonia emissions (−145,857 kg), slurry production (−153,879 m^3^), nitrogen emissions (−1,409,951 kg), and overall greenhouse gas emissions (91,768,362 kg CO_2_eq). Additionally, the feed and water consumption were substantially decreased by 53,237,805 kg and 292,978,094 L, respectively, further lowering the sector’s environmental footprint. The study demonstrates the effectiveness of customized eradication strategies and advanced breeding practices in reducing the environmental impact of animal husbandry. These findings underscore the necessity for ongoing collaboration among scientists, policymakers, and industry stakeholders to develop and implement sustainable livestock production methods. The Hungarian experience provides valuable insights into how targeted interventions can simultaneously improve production outcomes and reduce the environmental burden in the swine industry.

## 1. Introduction

Between 2014 and 2022, Hungary successfully completed its eradication program for porcine reproductive and respiratory syndrome (PRRS), a reemerging viral disease that poses significant economic challenges to commercial pig production worldwide. The methodologies, professional experiences, and improvements in production parameters following the eradication have been extensively documented in several studies [1,2,3]. The key objectives of the PRRS eradication program included the following: (1) enhance competitiveness through the adoption of modern, high-performance breeds; (2) improve production efficiency; (3) achieve freedom from multiple economically impactful infectious diseases; (4) strengthen biosecurity measures in Hungarian pig farming; (5) regulate live pig transport to prevent disease spread; and (6) promote healthier pork production to meet rising consumer demands, with a particular focus on reducing antibiotic use.

In Hungary, the PRRS eradication program primarily utilized the full herd replacement method (complete depopulation–repopulation). Over the 8-year program, more than 70,000 sows were replaced, accounting for approximately 35% of the total pig population. This approach led to a significant increase in the prevalence of higher genetic value breeds that were not only free from PRRS but also from other economically detrimental diseases. By 2022, the final year of the program, Hungary’s sow population had decreased by 26.2% compared to 2014 (from 200,200 to 147,800); however, the number of slaughter pigs produced only declined by 2.5% (from 3,959,287 to 3,860,616). Importantly, production efficiency—measured by the average number of slaughter pigs produced per sow per year—increased by over 32%, rising from 19.78 in 2014 to 26.12 in 2022 (Table 1) [2]. This improvement demonstrates the success of the program in maintaining high output despite a reduced sow population.

In recent years, the environmental impacts of animal production have attracted growing global attention, largely due to rising meat consumption. Meat consumption tends to increase significantly until a certain GDP per capita is reached (USD 36,000–45,000), after which it either stabilizes or declines; however, only a few countries have attained such high income levels. As a result, global meat consumption is expected to continue increasing. Among meat-producing animals, poultry and pork are the most resource-efficient and have the lowest environmental impact, whereas sheep and beef production are less favorable due to their higher water requirements and the methane emissions generated through rumination [4,5].

Life cycle assessment (LCA) is a tool used to comprehensively model the environmental impacts of food production. LCA evaluates the impact of a product or service on the environment throughout the entire production chain, identifying key points where emissions can be reduced. In the pig sector, which involves a complex production system, assessing environmental impacts requires consideration of various factors, including feed production, transport, animal husbandry, and waste management (e.g., manure and carcasses) [6].

Regardless of the animal species or production objectives, the lowest resource demand and environmental impact are achieved with high-performance breeds in precision systems [7]. In contrast, the highest resource demand and environmental impact are associated with extensive husbandry practices, particularly in free-range systems [8]. According to the European Environmental Protection Agency’s 2017 report, agriculture is responsible for 94% of total ammonia emissions from human activities across the EU, with 75% of these emissions resulting from manure management. Of the total ammonia emissions, cattle contribute 53%, pigs 25%, poultry 15%, and other animals (e.g., fish) 7% [6].

The intensification of pig farming has resulted in significant ammonia emissions. Pigs inefficiently convert the protein and other nitrogen forms in their feed into meat, excreting 50–80% of the nitrogen as waste. As a result, pig production is a major contributor to ammonia emissions from livestock. Key factors influencing ammonia release include manure moisture, nitrogen content, pH, temperature, aeration, and microbial activity [8].

When assessing the environmental impacts of animal production, it is essential to consider factors beyond the animals themselves. For instance, only 2% of the water used in animal production is consumed directly by the animals, with the vast majority being used to grow feed crops. Additionally, livestock production is a significant contributor to greenhouse gas emissions, accounting for approximately 15% of human-induced carbon dioxide emissions and driving substantial increases in methane production in recent decades [4].

Given these considerations, it is crucial to evaluate how the herd replacements during the PRRS eradication program have influenced environmental efficiency, the reduction in environmental load due to the introduction of high-performance breeds, and the overall changes in the ecological footprint (carbon footprint) of the Hungarian pig sector.

## 2. Materials and Methods

In Hungary, the procedures for environmental impact assessment and integrated pollution prevention and control (IPPC) permitting for pig farms are governed by Hungarian Government Decree No. 314/2005 (XII. 25.) [9], in line with Directive 2010/75/EU of the European Parliament and of the Council. According to this decree, pig farms with a capacity of at least 2000 places for pigs over 30 kg or 750 places for sows are required to undergo an environmental impact assessment as part of the IPPC requirements. Section 20/A of the decree mandates that the conditions and requirements specified in the permit be reviewed at least every 5 years, in accordance with the environmental protection regulations.

In this research, we analyzed IPPC reports from three large-scale Hungarian pig farms [10,11,12], using generally accepted standards for evaluating the impacts on air and water quality, waste management, soil, noise, the environment, wildlife, and landscape as a starting point [13]. The objective of this study was to assess the reduction in environmental pollution resulting from changes in the number of sows, assuming the same total weight and number of slaughter pigs. The data for sows were defined as follows:

- Ammonia (NH_3_) emission: 3 kg/sow/barn capacity/year.
- Slurry production (calculated with 9.5 months gestation and 2.5 months lactation): 9.5 × 2.2 + 2.5 × 0.43 = 3.165 m^3^/animal/year.
- Odor emission: 949 European odor unit (EOU). EOU is the amount of odor that, when evaporated in 1 m^3^ of neutral gas, causes the same physiological response from a panel (regarding detection threshold) as an EROM (European Reference Odor Mass) evaporated in 1 m^3^ of neutral gas. One EROM is equivalent to 123 µg of n-butanol (CAS 71-36-3), which, when evaporated, results in a concentration of 0.040 µmol/mol in 1 m^3^ of neutral gas.
- Nitrogen emission: 29 kg/sow/year [14].

Based on a study by Flachowsky [15], we calculated the ecological footprint or carbon footprint (CF) associated with operating with a reduced number of sows. The CF includes all greenhouse gas emissions that impact the climate, expressed in carbon dioxide equivalents (CO_2_eq). In this context, 1 unit of CO_2_eq corresponds to CO_2_, 23 units to CH_4_, and approximately 300 units to N_2_O. CO_2_eq is expressed in grams or kilograms per kilogram of product. The global average is approximately 3.5 kg CO_2_eq per kilogram of live weight (LW), but this can vary significantly, ranging from 1.7 to 9.3 kg CO_2_eq per kilogram of LW, depending on factors such as activity levels, accounting methods, specific technologies, and geographical conditions [16,17,18]. The current CO_2_eq benchmark adopted by the 27 EU member states for pig production is 7.55 kg CO_2_eq per kilogram of pork meat, although this may vary depending on the final product type [13].

According to pig production guidelines, the annual feed consumption per sow, based on an average intake of 3 kg/day, is 1095 kg [19]. The annual water consumption per sow, considering 2.2 reproductive cycles and 66 days of lactation per year, is 6026 L in total, derived from 66 days at 22.71 L/day and 299 days at 15.14 L/day [20].

## 3. Results and Discussion

As shown in Table 2, the environmental impact of pig production in Hungary largely decreased from 2014 to 2022, primarily due to complete herd replacements and enhanced production efficiency achieved through the PRRS eradication program.

Between 1985 and 2021, Hungary significantly reduced its greenhouse gas emissions, including CO_2_, CH_4_, N_2_O, SF_6_, NF_3_, perfluorocarbons, and fluorinated hydrocarbons (freons), from 117.1 million tons of CO_2_eq to 79.5 million tons of CO_2_eq (Table 3). Following the political transition in 1989, gross added value decreased across all economic sectors, including energy and agriculture, both of which are major sources of greenhouse gas emissions. From 1990 to 2019, Hungary’s per capita greenhouse gas emissions consistently remained below the EU28 average, reaching only 82% of the average in 2019 [21]. As of 2020, Hungary had 9,917,344 permanent residents [22], and the total national and household greenhouse gas emissions amounted to 76.8 million tons of CO_2_eq. This corresponds to a per capita greenhouse gas production of 7.74 tons of CO_2_eq.

By 2022, following the successful completion of the PRRS eradication program in Hungarian pig herds, many herds became free of several infectious diseases, primarily due to the whole herd replacement method (depopulation–repopulation). These herds not only achieved better health status but also exhibited improved genetic value, enhanced performance, increased fertility, and overall greater profit-generating capacity and efficiency. As a result of the program, the Hungarian pig industry was able to produce nearly the same number of slaughter pigs in 2022 as in 2014, with 52,400 fewer sows. The economic impact of this outcome was documented by Szabó et al. [2].

It is essential to distinguish between a disease-free animal (or herd) and a disease-resistant animal (or herd), as these are two separate concepts. A disease-free pig, even with the same genetic background, demonstrates better production capacity compared to its infected counterparts. This improved productivity results from the absence of infections [23] rather than resistance to specific pathogens, meaning the animal has not been genetically modified (naturally or artificially) for disease resistance. In this study, we focused on the results of full herd replacement with pigs that were free from the PRRS virus (PRRSV) but remained susceptible to it, while also possessing superior genetic traits that enhanced production efficiency.

Our research highlighted that the increased efficiency in pig production substantially reduced the sector’s environmental impact and pollution (Table 2). This improvement is not only reflected in the reduced slurry production due to fewer sows but also in the broader reduction in greenhouse gas emissions. Data indicate that between 2014 and 2020, while the overall greenhouse gas emissions from Hungarian agriculture increased (Table 3), the pig sector achieved a decrease of 91,768 tons of CO_2_eq. Although this reduction accounts for just 0.93% of the total CO_2_eq emissions from Hungarian agriculture, it is equivalent to the average annual greenhouse gas emissions (in CO_2_eq) of 11,856 Hungarian citizens.

Additionally, by the end of the PRRS eradication program, the pig sector’s feed consumption had decreased by more than 53,000 tons, and water usage had been reduced by nearly 300 million liters (Table 2), further contributing to the reduction in the sector’s environmental impact.

An important lesson from the Hungarian PRRS eradication program is that there is no universal method applicable to every pig farm to achieve successful results. Eradication strategies must be tailored to the specific conditions of each farm, considering all aspects of housing, feeding, rearing, and management—from vaccination and monitoring to the adaptation of farm technology.

The assertion that there are no one-size-fits-all solutions for pig farming aligns with the findings of Austrian researchers [24], who emphasized that environmental footprint analysis must be conducted at the national level with thorough and continuous evaluations (e.g., using the LCA method). To effectively reduce emissions, it is crucial to have a precise understanding of the entire production process within the sector. For instance, while Austrian pig feeds are primarily composed of locally sourced products like corn and wheat, the environmental impact in Spain is significantly influenced by the transportation of soybeans from Latin America, leading to differing environmental impact values and necessitating different interventions [24].

A study by Spanish authors [25] found that increasing the distance of feed transportation routes (from 400 km to 950 km and 1800 km) resulted in a fivefold increase in environmental impact at 950 km and a ninefold increase at 1800 km. The same study also used simulations to determine that reducing the slaughter weight (from 110 kg to 100 kg and 90 kg) decreases environmental impact, with breeding lighter pigs lowering the environmental burden by 11%.

## 4. Conclusions

The PRRS eradication program in Hungary, implemented between 2014 and 2022, demonstrated that full herd replacement combined with the introduction of high-performance breeds can significantly enhance both production efficiency and environmental sustainability in the swine sector. The program successfully reduced the sow population by 26.2% while maintaining nearly the same number of slaughter pigs, leading to substantial decreases in ammonia emissions (−145,857 kg), slurry production (−153,879 m^3^), nitrogen emissions (−1,409,951 kg), and overall greenhouse gas emissions (91,768,362 kg CO_2_eq). In addition, feed and water consumption were significantly reduced by 53,237,805 kg and 292,978,094 L, respectively, further contributing to the sector’s lower environmental footprint. These outcomes highlight the critical importance of tailored eradication strategies and advanced breeding practices in reducing the environmental impact of animal husbandry. The findings also underscore the need for ongoing collaboration among scientists, policymakers, and industry stakeholders to identify and implement the most effective methods for sustainable livestock production.

## Figures and Tables

**Table 1:** Total number of pigs produced and slaughtered in Hungary, and the number of slaughter pigs per sow per year (2014–2022) [2].

Year	Slaughter Pigs Produced	Sows *	Slaughter Pigs per Sow *
	Head		
2014	3,959,287	200,200	19.78
2015	3,818,906	196,800	19.41
2016	3,852,026	177,400	21.71
2017	3,586,603	171,500	20.91
2018	3,813,963	177,900	21.44
2019	3,867,987	155,300	24.91
2020	3,940,501	163,600	24.09
2021	4,032,407	156,900	25.70
2022	3,860,616	147,800	26.12

* Statistical analysis (*p* = 0.0002 for sows and *p* = 0.0001 for slaughter pigs per sow) revealed a highly significant association between the year and these variables.

**Table 2:** Reductions in environmental impact of swine production in Hungary (2014–2022).

Parameter	International Standard	Total in 2014 *	Total in 2022 *	Reduction (2014–2022) *
Ammonia (NH_3_) emissions	3 kg/sow/barn capacity/year	600,600 kg	454,753 kg	145,857 kg
Slurry production	3.165 m^3^/animal/year	633,633 m^3^	479,754 m^3^	153,879 m^3^
Odor emissions	949 European odor unit (EOU)	189,989,800 EOU	143,850,369 EOU	46,139,431 EOU
Nitrogen emissions	29 kg/sow/year	5,805,800 kg	4,395,849 kg	1,409,951 kg
Greenhouse gas emissions (Carbon footprint)	7.55 kg CO_2_eq/kg pork meat	377,877,500 kg CO_2_eq	286,109,138 kg CO_2_eq	91,768,362 kg CO_2_eq
Feed consumption	1095 kg/sow/year	219,219,000 kg	165,981,195 kg	53,237,805 kg
Water use	6026 L/sow/year	1,206,405,200 L	913,427,106 L	292,978,094 L

* In 2014, 200,200 sows were needed to produce 3,959,287 pigs for slaughter (Table 1). By 2022, thanks to the improved production efficiency achieved through the PRRS eradication program, the same number of slaughter pigs could have been produced with just 151,581 sows, reflecting a reduction of 48,619 sows over the 8-year period.

**Table 3:** Greenhouse gas emissions in Hungary (1985–2021) [21].

Year	Total	National Economy	Household	Agriculture, Forestry, and Fishery	Agriculture as % of National Economy	Agriculture as % of Household
	Thousand Tons of CO_2_ Equivalent					
1985	117,079	93,384	23,696	15,817	16.9	66.7
1986	114,726	91,720	23,005	15,475	16.9	67.3
1987	115,382	91,411	23,972	15,668	17.1	65.4
1988	110,552	86,160	24,392	15,225	17.7	62.4
1989	107,599	84,261	23,338	14,715	17.5	63.1
1990	100,785	76,359	24,426	12,932	16.9	52.9
1991	94,670	70,578	24,093	11,067	15.7	45.9
1992	84,852	64,363	20,488	9324	14.5	45.5
1993	85,920	64,667	21,253	8368	12.9	39.4
1994	84,794	65,283	19,511	8191	12.5	42.0
1995	83,936	65,096	18,840	7875	12.1	41.8
1996	86,037	67,185	18,853	8108	12.1	43.0
1997	84,497	66,337	18,160	8092	12.2	44.6
1998	84,051	67,466	16,585	8235	12.2	49.7
1999	84,590	67,453	17,137	8424	12.5	49.2
2000	84,193	64,793	19,400	8164	12.6	42.1
2001	86,649	65,558	21,091	8208	12.5	38.9
2002	82,480	63,620	18,859	8338	13.1	44.2
2003	87,208	64,908	22,300	8136	12.5	36.5
2004	86,863	65,790	21,073	8229	12.5	39.0
2005	88,512	66,141	22,371	7939	12.0	35.5
2006	87,677	65,424	22,253	7968	12.2	35.8
2007	87,567	65,937	21,629	7905	12.0	36.5
2008	85,695	64,434	21,261	8042	12.5	37.8
2009	82,200	58,621	23,579	7546	12.9	32.0
2010	83,828	59,593	24,235	7639	12.8	31.5
2011	83,306	58,929	24,377	7914	13.4	32.5
2012	78,415	53,803	24,612	7677	14.3	31.2
2013	76,770	52,812	23,958	8498	16.1	35.5
2014	76,472	54,224	22,248	8946	16.5	40.2
2015	80,609	56,474	24,135	9261	16.4	38.4
2016	80,449	56,063	24,386	9645	17.2	39.6
2017	82,666	58,247	24,419	9514	16.3	39.0
2018	81,793	58,959	22,834	9642	16.4	42.2
2019	80,279	57,524	22,755	9666	16.8	42.5
2020	76,761	54,623	22,138	9821	18.0	44.4
2021	79,502	55,633	23,869	9841	17.7	41.2

## Data Availability

The data supporting the findings of this study are included within the article. Further inquiries can be directed to the corresponding author.
